# Genome Wide Mapping of NR4A Binding Reveals Cooperativity with ETS Factors to Promote Epigenetic Activation of Distal Enhancers in Acute Myeloid Leukemia Cells

**DOI:** 10.1371/journal.pone.0150450

**Published:** 2016-03-03

**Authors:** Ryan P. Duren, Seth P. Boudreaux, Orla M. Conneely

**Affiliations:** 1 Program in Integrative Molecular and Biomedical Sciences, Baylor College of Medicine, Houston, Texas, United States of America; 2 Department of Molecular and Cellular Biology, Baylor College of Medicine, Houston, Texas, United States of America; Pohang University of Science and Technology (POSTECH), REPUBLIC OF KOREA

## Abstract

Members of the NR4A subfamily of orphan nuclear receptors regulate cell fate decisions via both genomic and non-genomic mechanisms in a cell and tissue selective manner. NR4As play a key role in maintenance of hematopoietic stem cell homeostasis and are critical tumor suppressors of acute myeloid leukemia (AML). Expression of NR4As is broadly silenced in leukemia initiating cell enriched populations from human patients relative to normal hematopoietic stem/progenitor cells. Rescue of NR4A expression in human AML cells inhibits proliferation and reprograms AML gene signatures via transcriptional mechanisms that remain to be elucidated. By intersecting an acutely regulated NR4A1 dependent transcriptional profile with genome wide NR4A binding distribution, we now identify an NR4A targetome of 685 genes that are directly regulated by NR4A1. We show that NR4As regulate gene transcription primarily through interaction with distal enhancers that are co-enriched for NR4A1 and ETS transcription factor motifs. Using a subset of NR4A activated genes, we demonstrate that the ETS factors ERG and FLI-1 are required for activation of NR4A bound enhancers and NR4A target gene induction. NR4A1 dependent recruitment of ERG and FLI-1 promotes binding of p300 histone acetyltransferase to epigenetically activate NR4A bound enhancers via acetylation at histone H3K27. These findings disclose novel epigenetic mechanisms by which NR4As and ETS factors cooperate to drive NR4A dependent gene transcription in human AML cells.

## Introduction

The NR4A subfamily of orphan nuclear receptors (*NR4A1*, *NR4A2*, *NR4A3*) are products of immediate early genes whose expression is induced by a variety of extracellular signals such as inflammatory cytokines, genotoxic stress, and apoptotic and mitogenic stimuli [1]. Activation of the NR4As results in transcriptional regulation of an overlapping set of target genes through monomeric interaction with a single NR4A response element (NBRE: AAAGGTCA) or through NR4A homo- and heterodimeric interaction with a NurRE (TGATATTTX6AAAGTCCA). NR4A2 has also been reported to interact indirectly with DNA via tethering to NFkB which results in suppression of NFkB target genes [2]. NR4A transcriptional activity is required for the majority of their characterized cellular functions [3–6], but NR4As also perform transcriptional activity-independent functions including DNA double stranded break repair [7] and promotion of apoptosis via interaction with BCL2 in the endoplasmic reticulum or mitochondria [8,9].

In the hematopoietic system, NR4As are important regulators of hematopoietic stem cell homeostasis [10,11] and play central roles in monocyte differentiation and T cell development [12,13]. Null mutation of both NR4A1 and NR4A3 results in a cell-autonomous and transplantable acute myeloid leukemia (AML)-like disease characterized by proliferative expansion of hematopoietic stem cells and accumulation of undifferentiated myeloid blasts in non-hematopoietic tissues [11]. Furthermore, reduced expression of NR4A1 and NR4A3 below a critical threshold in mice results in chronic myelodysplastic/myeloproliferative disease (MDS) [14]. Importantly, retroviral rescue of NR4A1 or NR4A3 expression after AML development is sufficient to deplete AML leukemia initiating cells (LICs) in vivo in a competitive transplant setting in mice [15].

In humans, NR4A expression is reduced in MDS patients [16,17] and silenced in human AML blasts, regardless of patient cytogenetics or French-American-British classification [11,15,18]. Rescued expression of either NR4A1 or NR4A3 in several cytogenetically distinct human AML cell lines inhibits cell viability and long term proliferation in vitro. By profiling gene signatures that are regulated by NR4As in Kasumi-1 AML cells (carrying the AML-ETO oncogene), we have shown that NR4As reprogram a subset of gene signatures that distinguish primary human LICs from normal hematopoietic stem cells (HSCs), which includes suppression of a core MYC oncogenic program through direct repression of MYC expression [15]. Most importantly, NR4A transcriptional capability is absolutely required for their tumor suppressive functions. These observations indicate that the tumor suppressive capability of NR4As may extend to human AML and that reactivation of these genes could provide therapeutic benefits in the treatment of AML. However, the transcriptional mechanisms and direct target genes through which NR4As exert their cell selective functions remain poorly understood.

Here, we have used chromatin immunoprecipitation followed by high throughput sequencing (ChIP-seq) to identify genome wide NR4A binding sites, and we have integrated these data with gene expression signatures that are acutely regulated upon rescue of NR4A expression in human AML cells to identify direct genomic targets of NR4A1 and investigate its global transcriptional mechanisms of action. Using this integrated unbiased approach, we reveal novel cooperative interactions between NR4As and members of the ETS family of transcription factors to promote transcription of NR4A target genes. We show that NR4As can reprogram ETS factor binding to NR4A bound enhancers to promote p300 histone acetyl transferase (HAT) recruitment and epigenetic enhancer activation via p300 dependent acetylation of histone H3 at lysine 27 (H3K27Ac).

## Materials and Methods

### Cell culture, plasmids, and mRNA in vitro transcription

Kasumi-1 cells were purchased from ATCC. Cells were maintained in 1640 RPMI with 20% FBS. The plasmids and *In-vitro* transcribed RNA *(IVT-RNA)* transfection system have been previously described [15]. *In-vitro* transcription (IVT) was performed per manufacturer’s instructions on linearized plasmid containing the NR4A1 coding sequence using the mMESSAGE mMACHINE^®^ T7 Kit (Applied Biosystems). RNA polyadenylation was performed with a Poly(A) Tailing Kit, and resulting IVT-RNA was purified with a MEGA Clearance Kit (Applied Biosystems) according to manufacturer instructions. For electroporation, cells were suspended to a final concentration of 1 million cells per 100uL DPBS. 200uL of cell solution was transferred to 0.4 cm cuvettes (USA Scientific), mixed by pipetting with IVT-RNA at a final concentration of 100 nM, and immediately electroporated at 330V for 5 milliseconds with the GenePulser Xcell system (Bio-Rad).

### RT- and real time-qPCR

Total RNA was extracted using RNeasy Mini Kit (Qiagen), and RNA was reverse transcribed into cDNA using High Capacity cDNA Reverse Transcription Kits (Applied Biosystems). cDNA was diluted 5 fold with nuclease-free water and quantitated via TaqMan Gene Expression Assays and Master Mix (Applied Biosystems, Carlsbad, California). TaqMan pre-designed primers and probes (Applied Biosystems) were used for target gene qPCR. PCR amplifications were performed using the ABI Step One Plus Sequence Detection System (Applied Biosystems) under standard conditions. Transcript levels were determined by standard curve and normalized to corresponding *β2m* levels.

### Microarray analysis and bioinformatics

Total RNA was extracted 6h after IVT transfection using an RNeasy Mini kit (Qiagen). Quality control and processing of human genome U133 Plus 2.0 (Affymetrix, Santa Clara, CA) chips were performed by the Baylor College of Medicine Genomic and RNA Profiling Core. Protocols from the Affymetrix GeneChip Expression Analysis Technical Manual were used for preparation and fragmentation of biotin labeled cRNA. The Affymetrix GeneChip Fluidics Station 400 was used to perform array hybridization, washing, and staining with streptavidin-phycoerythrin. Probe fluorescence values were normalized by Robust Multiarray Analysis (RMA) using RMA Express. Differentially expressed probes were identified by Rank Product Analysis in the TM4 Microarray Software Suite [19,20] and considered significant with a q-value ≤0.05. GO annotation was performed on significantly regulated genes using DAVID Bioinformatics Resources [21,22], and Gene Set Enrichment Analysis was performed on all array probes with 1000 permutations [23,24].

### ChIP-sequencing (Illumina)

4hr after NR4A1 IVT transfection, duplicate samples of 30 million Kasumi-1 cells were fixed with 1% formaldehyde for 15 minutes at room temperature and then quenched with 0.125M glycine for 5 minutes. Cells were washed twice with cold DPBS and frozen at -80°C. ChIP-sequencing was performed by Active Motif, Inc. Cells were lysed by Dounce homogenization and chromatin was sheared to an average length of 300–500bp. 30ug aliquots of lysate were pre-cleared with Protein A agarose beads and incubated with 2ug NR4A antibody (sc-990, Santa Cruz Biotechnology) overnight at 4°C. Immune complexes were captured with Protein A agarose beads for 3 hours and washed with low salt, high salt, LiCl, and TE buffers. Immune complexes were eluted with SDS buffer, and subjected to RNase treatment and proteinase K treatment. Crosslinks were reversed by incubation overnight at 65°C, and ChIP DNA was purified by phenol-chloroform extraction and ethanol precipitation. ChIP DNA was amplified by following the Illumina ChIP-Seq DNA Sample Prep Kit protocol. DNA libraries were quantified and sequenced on a Genome Analyzer II using 36nt single end reads.

### ChIP-seq data analysis

Sequences were aligned to the human genome (NCBI37/hg19) using the BWA algorithm. Alignments were extended in silico at their 3’ ends to a length of 150bp and assigned to 32nt bins. Peak locations were determined using the MACS (Model based analysis of ChIP-seq) algorithm with a p-value cutoff of 1E-10 [25], and were annotated to the nearest gene within 100kb using Active Motif’s proprietary Genpathway software. ChIP-seq tracks were visualized in the UCSC genome browser [26]. NR4A1 binding regions were integrated with microarray data by assigning each peak location to the TSS of the nearest regulated gene. Motif discovery was performed on the 300bp sequence surrounding the center of each NR4A1 peak using SeqPos [27]. The Cistrome Analysis Pipeline was used to generate ChIP-seq peak correlation plots and for intersection of NR4A1 genomic coordinates with published datsatets [28]. Regions that shared at least 1 nucleotide were considered overlapping for all analyses.

### ChIP-qPCR

Chromatin immunoprecipitations were performed 4h after IVT electroporation. Cells were crosslinked with 0.75% formaldehyde for 10min at room temperature and quenched with 0.125M glycine for 5min. Cells were washed twice with cold DPBS, and cell pellets containing 5 million cells were re-suspended in 200ul FA Lysis Buffer (50 mM HEPES-KOH pH7.5, 140 mM NaCl, 1 mM EDTA pH8, 1% Triton X-100, 0.1% Sodium Deoxycholate, 0.1% SDS). Cell lysate was sonicated for 60min using a BioRuptor (Diagenode, Inc.) with a 30sec on, 30sec off cycle in wet ice to achieve chromatin fragments of 100–300bp. For NR4A1, FLI-1, and p300 ChIPs, 50ug pre-cleared lysate was diluted 10-fold in ChIP dilution buffer (1% TritonX-100, 2mM EDTA, 20mM Tris-HCl, 150mM NaCl) and incubated with 4ug of appropriate antibody overnight. For histone modification ChIPs, diluted lysate was incubated with 2ug of appropriate antibody overnight at 4°C. Immune complexes were captured with Protein A/G agarose beads (Santa Cruz Biotechnology) and washed successively for 5min each at 4°C with Low Salt(1% Triton X-100, 150mM NaCl, 2mM EDTA, 20mM Tris-HCl), High Salt(1% Triton X-100, 500mM NaCl, 2mM EDTA, 20mM Tris-HCl), and LiCl(0.25M LiCl, 1% NP-40, 1% deoxycholate, 1mM EDTA, 10mM Tris-HCl) wash buffers followed by three TE buffer washes. Input and immunoprecipitated DNA crosslinks were reversed overnight at 65°C and DNA was purified with QiaQuick PCR Purification Kit (Qiagen). ChIP DNA was quantitated with Power SYBR Green Master Mix (Life Technologies) and normalized to input DNA. Antibodies used for ChIP were anti-NR4A (sc-990), anti-p300 (sc-585), anti-FLI1 (sc-356), and rabbit IgG (sc-2027) (Santa Cruz Biotechnology); anti-histone H3 (ab1791), anti-H3K9Ac (ab10812), anti-H3K27ac (ab4729), anti-H3K4me1 (ab8895), and anti-H3K4me3 (ab8580) (Abcam); and anti-H3K27me3 (9733S, Cell Signaling Technology). ChIP-qPCR primer sequences are listed in S1 Table.

### siRNA

Kasumi-1 cells were electroporated with 200nM of each appropriate siRNA or with equal amounts of non-targeting control siRNA (Dharmacon) 48h before electroporation with in vitro transcribed NR4A1 mRNA. RNA was extracted 4 hours or 6 hours after electroporation with NR4A1 IVT for ChIP and gene expression analysis, respectively, and processed as above.

## Results

### Identification of the NR4A1 targetome

**Fig 1:**
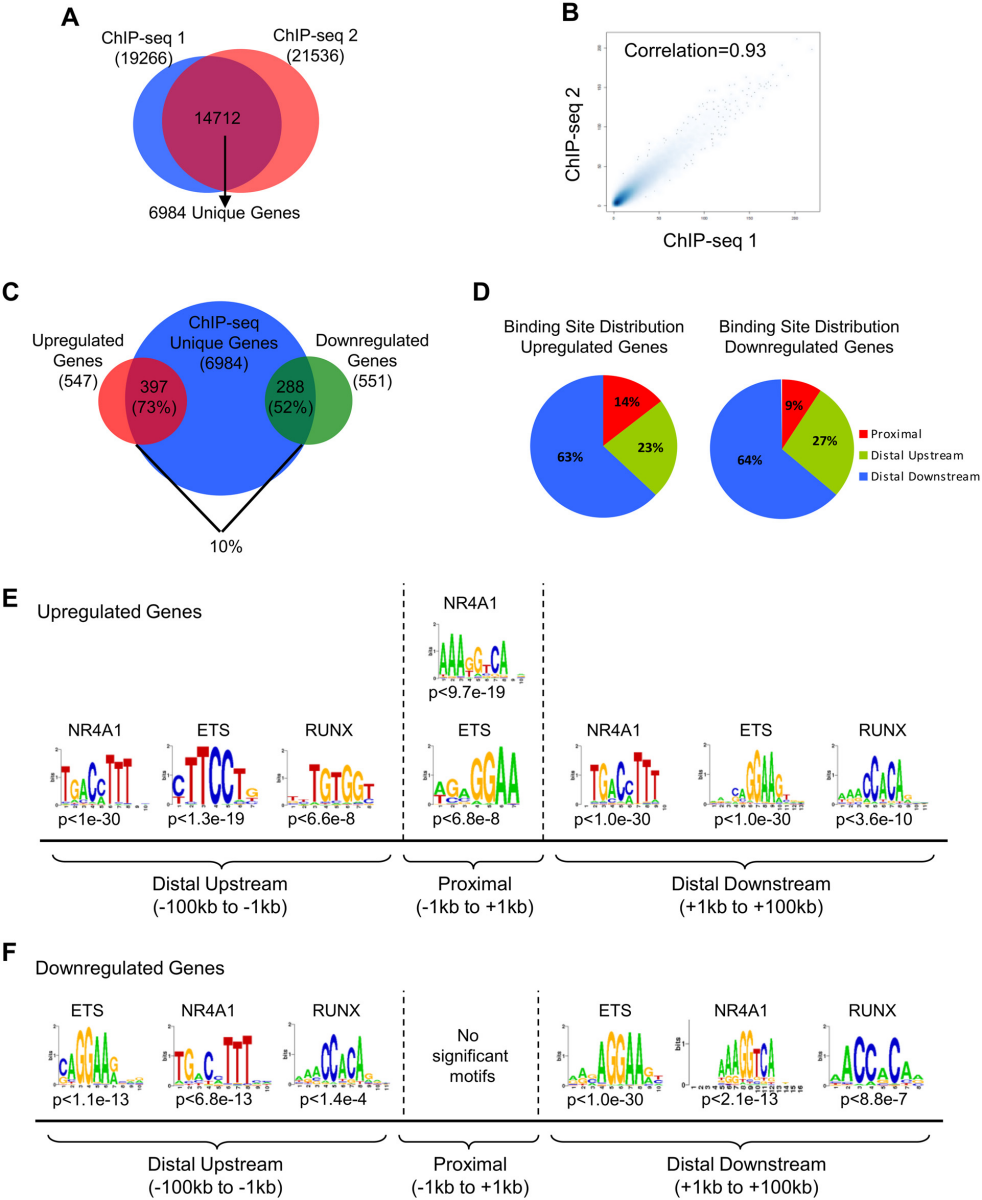
Identification of the NR4A1 targetome in Kasumi-1 AML cells. (**A**) Intersection of NR4A1 binding sites from duplicate ChIP-seq samples. Shared binding sites were defined as peak intervals that overlap by at least 1bp. (**B**) Correlation plot of NR4A1 peak heights from duplicate ChIP-seq samples. (**C**) Venn diagram representing integration of unique NR4A1-dependent upregulated (red) and downregulated (green) genes at 6hr with NR4A1 binding sites (blue) determined by ChIP-seq at 4hr. NR4A1 binding sites were assigned to the nearest regulated TSS. (**D**) NR4A1 binding site distribution at induced and repressed genes. (**E**,**F**) Seq-pos motif analysis of NR4A1 binding sites associated with induced (**E**) and repressed (**F**) genes.

We first sought to identify direct NR4A1 target genes through integration of NR4A1-dependent gene expression signatures with NR4A1 genome wide binding sites in Kasumi-1 AML cells. Since Kasumi-1 cells do not express endogenous NR4A1, NR4A2, or NR4A3 proteins, and we have previously demonstrated that NR4A1 and NR4A3 regulate redundant transcriptional programs, we introduced NR4A1 mRNAs via electroporation as a model of NR4A1 activity and used a well validated anti-NR4A antibody (sc-990, Santa Cruz) for ChIP-seq analysis of genome wide NR4A1 binding to chromatin. We have previously shown that this antibody is monospecific for reexpressed NR4A1 in this system [15]. Using the MACS model-based peak finding algorithm [25], ChIP-seq identified 19,266 and 21,536 NR4A1 interaction sites in two replicate cistromes when normalized to input within 4 hours of NR4A1 expression. Intersection of the two replicates resulted in ~70% binding site concordance and yielded 14,712 high confidence peaks representing 6,984 unique genes (Fig 1A). Peak height correlation between the overlapping binding sites (r = 0.91) indicated good reproducibility between the two replicate samples (Fig 1B). Furthermore, interrogation of the ChIP-seq data revealed reproducible binding sites associated with the proximal promoter region spanning the validated canonical NR4A binding site located -145bp upstream of the ENO3 transcription start site (TSS) (S1 Fig).

We have previously shown that NR4A-dependent growth repression requires the NR4A DNA binding domain (DBD), suggesting that classical DNA binding and transcriptional regulation is required for cell growth inhibition by NR4As [15]. To address the underlying mechanism, we first compared transcriptional profiles from expression of wild type NR4A1 (NR4A1^WT^) and NR4A1 with C284A and E285A mutations of a zinc finger within the DNA binding domain (NR4A1^CEAA^) that are essential for NR4A DNA binding [2]. Microarray-based gene expression profiling revealed that NR4A1^WT^ activates 547 genes and represses 551 genes within 6 hours of expression in Kasumi-1 cells (S2 Fig). Gene set enrichment analysis (GSEA) revealed repression of a core MYC oncogenic signature that was selective to NR4A1^WT^ and was not observed upon expression of the mutant NR4A1^CEAA^ (S2 Fig). Gene ontology annotation of upregulated genes by NR4A1^WT^ identified enrichment of genes associated with lymphocyte differentiation and apoptosis while ribosome biogenesis and cell proliferation were enriched annotations within the repressed genes (S2 Fig). Interestingly, while the NR4A1^CEAA^ mutant did not recapitulate the NR4A1^WT^ gene signature, NR4A1^CEAA^ was capable of transcriptional regulation of an entirely distinct cohort of 209 upregulated genes that were enriched for ribonucleotide binding, cell cycle, and proto-oncogenes and 239 downregulated genes that were enriched for small GTPase signal transduction, actin cytoskeleton organization, and endomembrane system (S2 Fig), consistent with our previous observation that DNA binding is required for NR4A-dependent tumor suppression [15].

To identify direct target genes associated with NR4A tumor suppression, we therefore, integrated NR4A1 binding sites with the nearest TSS regulated by NR4A1^WT^. This analysis revealed that a minority of genome-wide NR4A1 binding corresponded with NR4A1-dependent gene regulation in Kasumi-1 cells since only 10% of the 6982 unique genes bound by NR4A1 were transcriptionally regulated by NR4A1 (Fig 1C). Nevertheless, the majority (62%) of NR4A1 regulated genes were bound by NR4A1, and a larger number of upregulated genes (79%) were associated with NR4A1 binding as compared to downregulated genes (54%) (Fig 1C).

Consistent with other nuclear receptors [29–31], the majority of NR4A1 binding sites associated with regulated genes were located distal to the TSS (Fig 1D). Of binding sites associated with upregulated genes, 14% were found within proximal regions (+/- 1kb from TSS), 23% were distal upstream (-100kb to -1kb), and 63% were distal downstream (+1kb to +100kb) to the nearest TSS. In the case of NR4A1 downregulated genes, 9% of binding sites were found within proximal regions, 27% were distal upstream, and 64% were distal downstream (Fig 1D).

Motif analysis revealed that NR4A1-occupied regions at both upregulated and downregulated genes were highly enriched for the NR4A-responsive NBRE and ETS factor family motifs, while RUNX motifs were enriched to a much lesser extent (Fig 1E and 1F). The NR4A NBRE was identified as the most enriched motif in distal upstream and proximal regions of upregulated genes followed by ETS and then RUNX motifs. Only the ETS motif was enriched in addition to the NR4A NBRE in regions proximal to the TSS. Further, NR4A and ETS motifs were equally enriched in distal downstream regions of upregulated genes (Fig 1E). Motif analysis of repressed genes showed generally similar motif enrichment as activated genes with the exception of regions proximal to the TSS. Distal upstream regions of repressed genes were equally associated with NR4A and ETS motifs while distal downstream regions were most enriched for ETS motifs. However, proximal NR4A1 binding regions lacked significantly enriched motifs (Fig 1F). Together, these data suggest that NR4A1 may cooperate with ETS and, to a lesser degree, RUNX factors to regulate gene expression in AML cells. Furthermore, motif analysis predicts that NR4A1 can regulate target gene expression via direct binding to canonical NBREs and/or by interaction with ETS factors to activate or repress NR4A dependent gene signatures.

### Chromatin accessibility and ERG/FLI-1 occupancy predict distinct modes of transcriptional regulation by NR4A1

**Fig 2:**
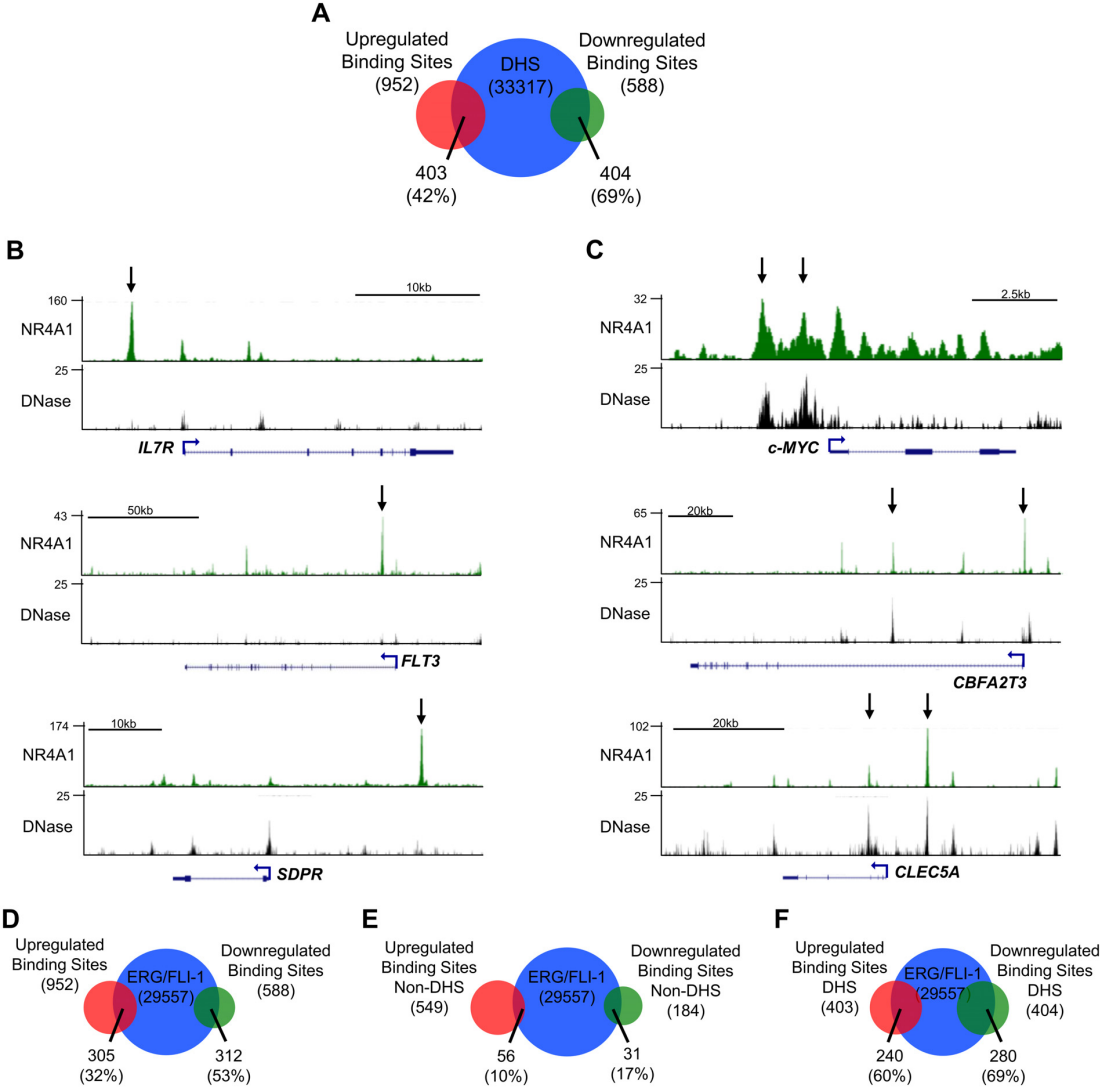
NR4A1 binding sites at induced and repressed genes display differential preference for DHS regions. (**A**) NR4A1 binding sites associated with induced (red) and repressed (green) genes within DHS regions (blue) from Kasumi-1 cells. (**B**,**C**) Examples of NR4A1 ChIP-seq occupancy (green) and DHS profiles (black) at genes upregulated (**B**) and repressed (**C**) by NR4A1. Arrows denote NR4A1 binding peaks without DHS at upregulated genes and NR4A1 binding peaks with DHS at repressed genes. The y axis represents cumulative tag counts at each region, and RefSeq transcripts are shown below. (**D**) Overlap of NR4A1 binding sites at upregulated (red) and downregulated (green) genes with ERG/FLI-1 binding sites identified in SKNO-1 cells (blue). (**E**,**F**) Venn diagram representing overlap between NR4A1 binding sites within non-DHS (**E**) or DHS (**F**) regions at induced (red) and repressed (green) genes and ERG/FLI-1 binding sites in Kasumi-1 cells.

DNaseI hypersensitivity (DHS) can be used to identify regions of chromatin accessibility [32], and motif analysis of DHS regions has been proposed as a method of identifying transcription factors that occupy those regions of DNA [33,34]. We predicted that we may distinguish the chromatin status of NR4A1 binding sites by integrating the NR4A1 targetome with a publically available genome wide DHS-seq dataset obtained in Kasumi-1 cells [35]. Intersection of these data revealed two apparently distinct modes of NR4A1 binding to chromatin. The majority of NR4A1 binding sites at induced genes (58%) were found in non-DHS regions, while in contrast, the majority (69%) of binding sites associated with repressed genes were located in DHS regions (Fig 2A). Examples of NR4A1 binding sites in non-DHS regions of induced genes include *IL7R*, *FLT3*, and *SDPR* (Fig 2B), and NR4A1 binding within DHS regions of repressed genes includes the proliferative genes *c-MYC CBFA2T3*, and *CLEC5A* (Fig 2C).

Given the high degree of ETS motif coenrichment within NR4A1 binding sites, we next asked whether NR4A1 and ETS factors cooperate at a genome wide level to regulate NR4A1 target genes. We queried published genome-wide binding data for the ETS factors ERG and FLI-1, two of the most highly expressed ETS factors in human AML-ETO positive AML cells [36], to determine whether NR4A1 may bind to regions of ERG or FLI-1 occupancy. This analysis revealed that 53% of NR4A1 binding sites associated with repressed genes are pre-occupied by ERG/FLI-1 while only 32% of NR4A1 sites associated with induced genes overlap with ERG/FLI-1 binding (Fig 2D). Finally, we integrated ERG/FLI-1 binding data with NR4A1 binding sites in both non-DHS and DHS regions of chromatin. We found that NR4A1 binding sites within non-DHS regions were not occupied by ERG/FLI-1 (Fig 2E) while NR4A1 bound sites within DHS regions were pre-occupied by ERG/FLI-1 (Fig 2F). Collectively, these data indicate that the majority of NR4A1 binding sites at upregulated genes reside in unoccupied genomic regions with relatively low chromatin accessibility, while the majority of NR4A1 binding sites at repressed genes reside in accessible DNaseI hypersensitive genomic regions pre-occupied by ERG/FLI-1.

### Opposing transcriptional regulation of lineage developmental and proliferative target genes by NR4A1

**Fig 3:**
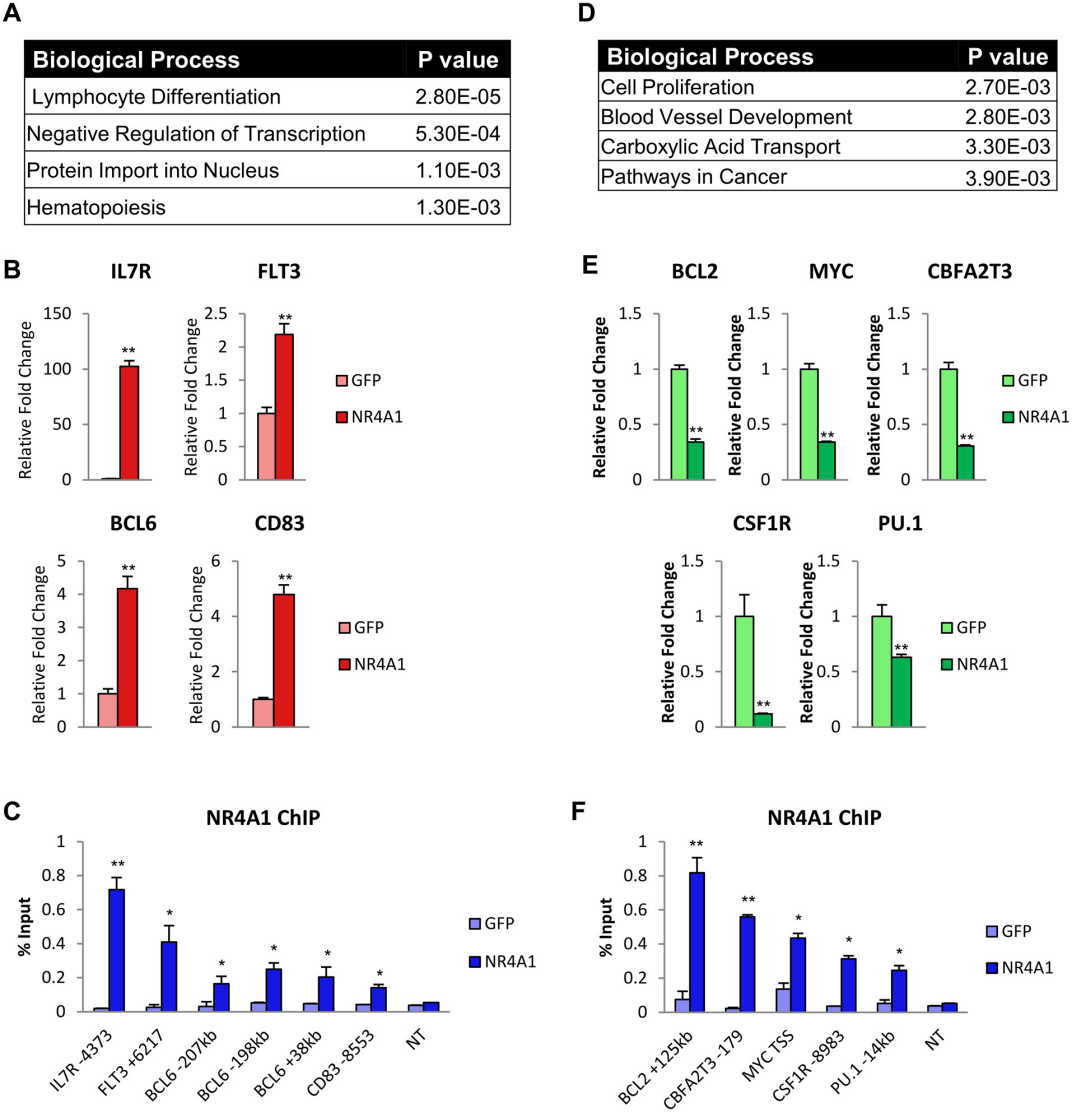
NR4A1 directly regulates expression of cell developmental and cellular proliferation genes. (**A**) Summary of enriched GO terms for directly upregulated genes using DAVID functional annotation. (**B**) RT-qPCR validation of NR4A1-dependent induced genes at 6hr (sample size = 3). (**C**) ChIP-qPCR analysis of NR4A1 recruitment to induced genes in Kasumi-1 cells at 4hr (sample size = 3). (**D**) Gene ontology annotations associated with repressed genes. (**E**) RT-qPCR validation of repressed genes at 6hr (sample size = 3). (**F**) ChIP-qPCR analysis of NR4A1 recruitment to repressed genes at 4hr in Kasumi-1 cells (sample size = 3). NT = non-transcribed region in a gene desert of chr7. Results are means ± s.d. **p<0.01, *p<0.05.

To gain insight into transcriptional programs regulated by NR4A1, we identified enriched gene ontology (GO) groups of induced and repressed direct target genes. Enriched GO annotations of induced genes included lymphocyte differentiation, negative regulation of transcription, protein import into the nucleus, and hematopoiesis. The most enriched biological process, lymphocyte differentiation (Fig 3A), included several of the most highly induced NR4A1 target genes such as *IL7R*, *FLT3*, and *BCL6* (S3 Fig), and we also noted direct NR4A1 upregulation of the dendritic cell surface marker *CD83* [37]. NR4A1-mediated induction of these genes was independently validated by qPCR (Fig 3B), and NR4A1 recruitment to distal regions of each of these induced genes (S3 Fig), was independently validated by ChIP-qPCR (Fig 3C).

Conversely, repressed direct target genes were enriched for GO annotations that included cell proliferation, blood vessel development, carboxylic acid transport, and pathways in cancer (Fig 3D). The cell proliferation GO annotation was most highly enriched in the directly repressed target genes and included the known hematopoietic oncogenes *c-MYC*, *BCL2*, *CBFA2T3*, and *CSF1R* (S4 Fig). Interestingly, NR4A1 also directly repressed the master myeloid transcription factor *PU*.*1* which regulates *CSF1R* dependent leukemic stem cell survival [38]. NR4A1-mediated transcriptional repression of a subset of these genes was validated by qPCR (Fig 3E), and recruitment of NR4A1 to elements associated with these genes (S4 Fig) was validated by ChIP-qPCR (Fig 3F).

### NR4A1 establishes an active enhancer landscape and distal binding regions of NR4A activated genes

**Fig 4:**
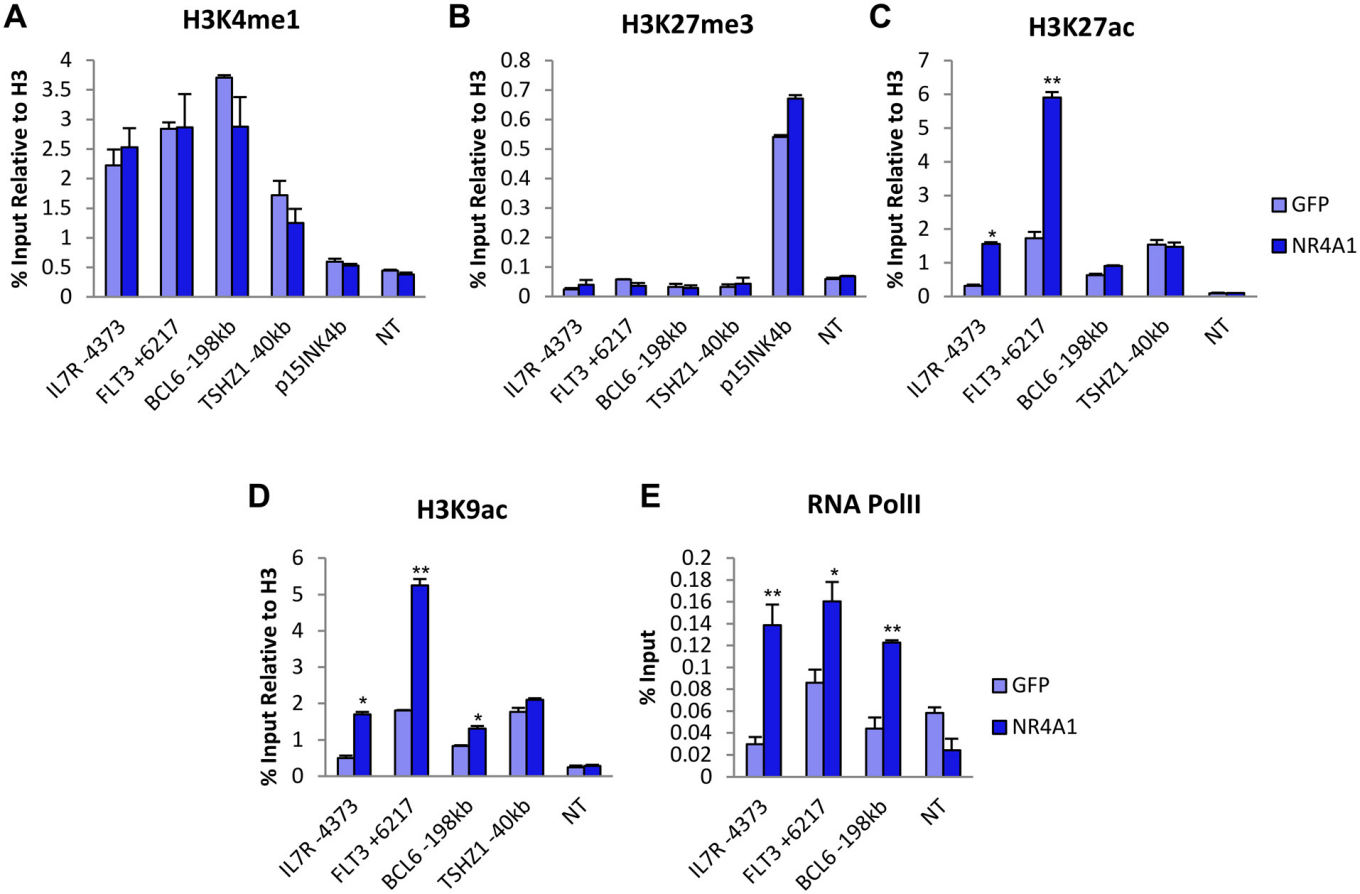
NR4A1 activates primed enhancers at induced genes. ChIP-qPCR analysis of (**A**) H3K4me1, (**B**) H3K27me3, (**C**) H3K27ac (**D**) H3K9ac and (**E**) Pol II at NR4A1 binding sites of induced genes in Kasumi-1 cells (sample size = 3). The *p15Ink4b* locus was used as positive control for H3K27me3, and *TSHZ1* was used as positive control for H3 acetylation. NT = non-transcribed region in a gene desert of chr7. Results are means ± SD. **p<0.01, *p<0.05.

The lack of overlap between NR4A1 binding sites and DHS regions of NR4A1 upregulated genes in Kasumi-1 cells predicted that NR4A1 binds distal enhancers of NR4A inducible genes via interaction with unoccupied chromatin. To address the epigenetic mechanisms by which NR4A1 regulates expression of these genes, we examined the epigenetic landscape of distal NR4A1 binding regions associated with a subset of upregulated genes by examining their histone modification status. Distal enhancer regions are generally associated with relatively high levels of histone monomethylation at histone H3K4 (H3K4me1) in contrast to H3K4me3 which reflects a common mark of active promoter elements [39,40]. The activation state of H3K4me1 marked enhancers is further distinguished by the presence or absence of additional histone marks, in particular the acetylation or methylation status of H3K27 (H3K27ac or H3K27me3). H3K4me1 primed but inactive enhancers lack H3K27ac, a mark that is acquired upon enhancer activation [41,42]. Further, a subset of H3K4me1+/H3K27ac-primed enhancers also contain high levels of H3K27me3, a mark associated with polycomb complex mediated repression [40,42]. We queried the status of each of these histone marks at NR4A1 distal binding regions of selected lymphoid genes. As a positive control for active enhancers, we used a distal upstream site associated with the *TSHZ1* gene that displays one of the highest levels of H3K9ac within a DHS region [35]. The *p15INK4b* locus, which is silenced in Kasumi-1 cells and is marked by H3K27me3[43], was used as a positive control for repressed chromatin. We found relatively high levels of H3K4me1 associated with the distal NR4A1 binding regions of *IL7R*, *FLT3* and *BCL6* relative to inactive control loci (Fig 4A), and this mark was unaffected by NR4A1. Interestingly, the same regions of all three genes lacked enrichment for H3K27me3 relative to p15Ink4B positive control and displayed relatively low H3K27ac in the absence of NR4A1 (Fig 4B and 4C). However, recruitment of NR4A1 to these regions was associated with marked NR4A1 dependent enrichment of H3K27ac and H3K9Ac at distal NR4A binding regions of both *IL7R* and *FLT3* and to a lesser degree at *BCL6* (Fig 4C and 4D). Consistent with NR4A1-dependent gene activation, NR4A1 also increased enrichment of RNA PolII to all binding sites associated with activated genes, including *BCL6* despite the lower NR4A dependent enrichment of H3K27ac or H3K9ac at this enhancer (Fig 4E). Taken together, these data indicate that NR4A1 can bind to H3K4me1 primed distal enhancer elements of a subset of NR4A induced genes to establish an active enhancer landscape by enabling histone acetylation at H3K27 and H3K9.

### NR4A1 recruits p300 to H3K4me1 primed enhancers of induced genes to promote p300 mediated enhancer acetylation and gene activation

H3K27 is a key substrate for acetylation by the highly homologous histone acetyl transferases (HATS), p300 and CBP [42,44,45]. These HATS are often recruited cooperatively by a wide range of TFs including NRs to facilitate transcription activation via histone acetylation of multiple lysines on a single histone [44,46]. Further, genome binding of the p300 HAT correlates with tissue specific enhancer activity [47] and can define enhancer landscapes more distinctly than histone modifications [48].

**Fig 5:**
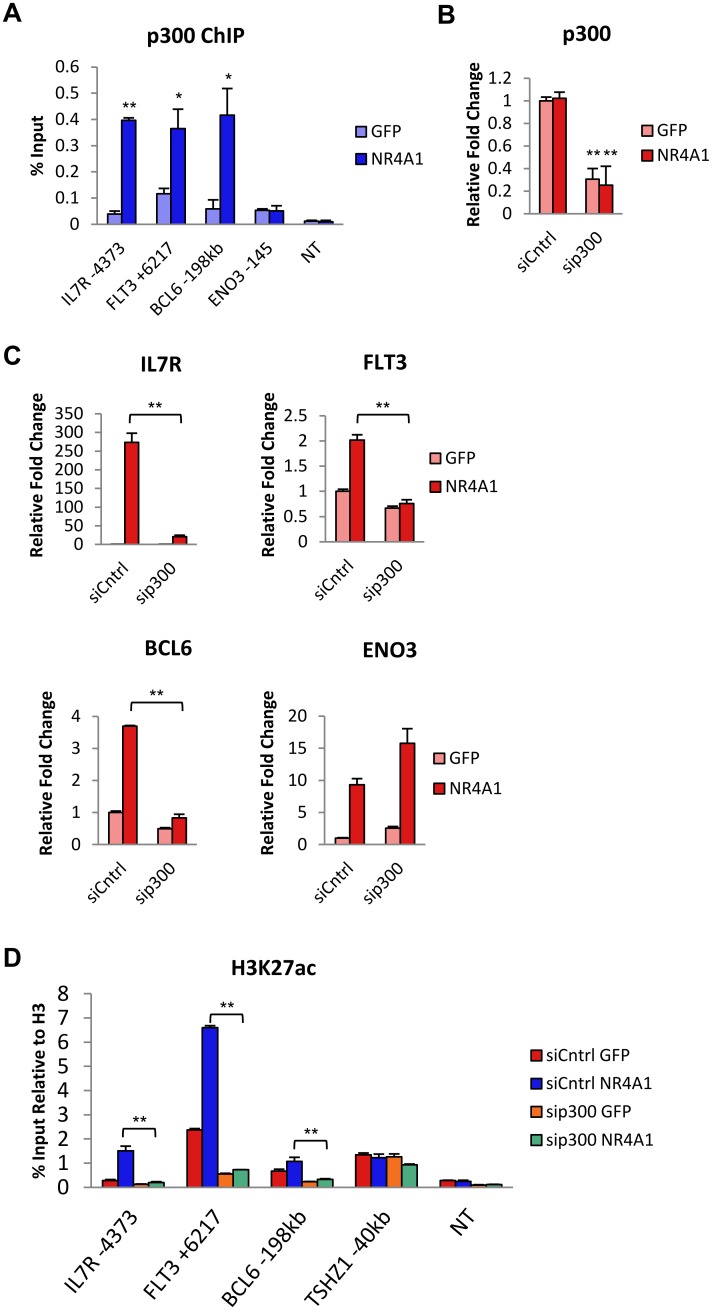
p300 is required for NR4A1-mediated enhancer activation and gene induction. (**A**) ChIP-qPCR of p300 binding at NR4A1 binding sites of upregulated genes at 4hr (sample size = 3). (**B**) RT-qPCR validation of siRNA-mediated knockdown of p300 at 48hr (sample size = 3). (**C**) RT-qPCR analysis of upregulated genes 48hr after p300 knockdown and 6hr after NR4A1 expression (sample size = 3). (**D**) ChIP-qPCR analysis of H3K27ac at NR4A1 binding sites after p300 knockdown for 48hr (sample size = 3). NT = non-transcribed region in a gene desert of chr7. Results are means ± SD. **p<0.01, *p<0.05.

To address the contribution of p300/CBP to NR4A1 dependent enhancer activation, we first asked whether NR4A1 was capable of recruiting p300 to activated enhancer elements using ChIP-qPCR of p300 at distal NR4A1 bound enhancers. This analysis revealed robust NR4A1 dependent recruitment of p300 to the distal NR4A1 binding sites of all activated genes tested (Fig 5A). Further, recruitment of p300 was selective for these distal elements since p300 recruitment was not observed at the proximal NR4A1 binding site of *ENO3* (Fig 5A). We next asked whether p300 recruitment by NR4A1 is required for NR4A1 dependent induction of gene expression using siRNA-mediated knockdown of p300. We confirmed efficient knockdown of p300 mRNA expression in Kasumi-1 cells at 48 hours after siRNA electroporation (Fig 5B) and found depletion of p300 was sufficient to potently inhibit NR4A1 dependent induction of *IL7R*, *FLT3* and *BCL6* (Fig 5C). However, as predicted by the lack of NR4A1 dependent recruitment of p300 to *ENO3*, NR4A1 dependent induction of *ENO3* was unaffected by depletion of p300 (Fig 5C). Finally, we asked whether NR4A1 dependent recruitment of p300 was required for acquisition of histone acetylation at poised enhancers of upregulated genes. As expected, depletion of p300 strongly abrogated NR4A1 dependent acetylation of H3K27 at NR4A1 binding sites (Fig 5D). Together, these data reveal that NR4A1 can bind to distal primed but inactive enhancer elements of a subset of NR4A induced target genes to promote enhancer activation and gene expression via recruitment of p300 to acetylate H3K27.

### ETS factors cooperate with NR4A1 to activate distal enhancers of induced target genes

**Fig 6:**
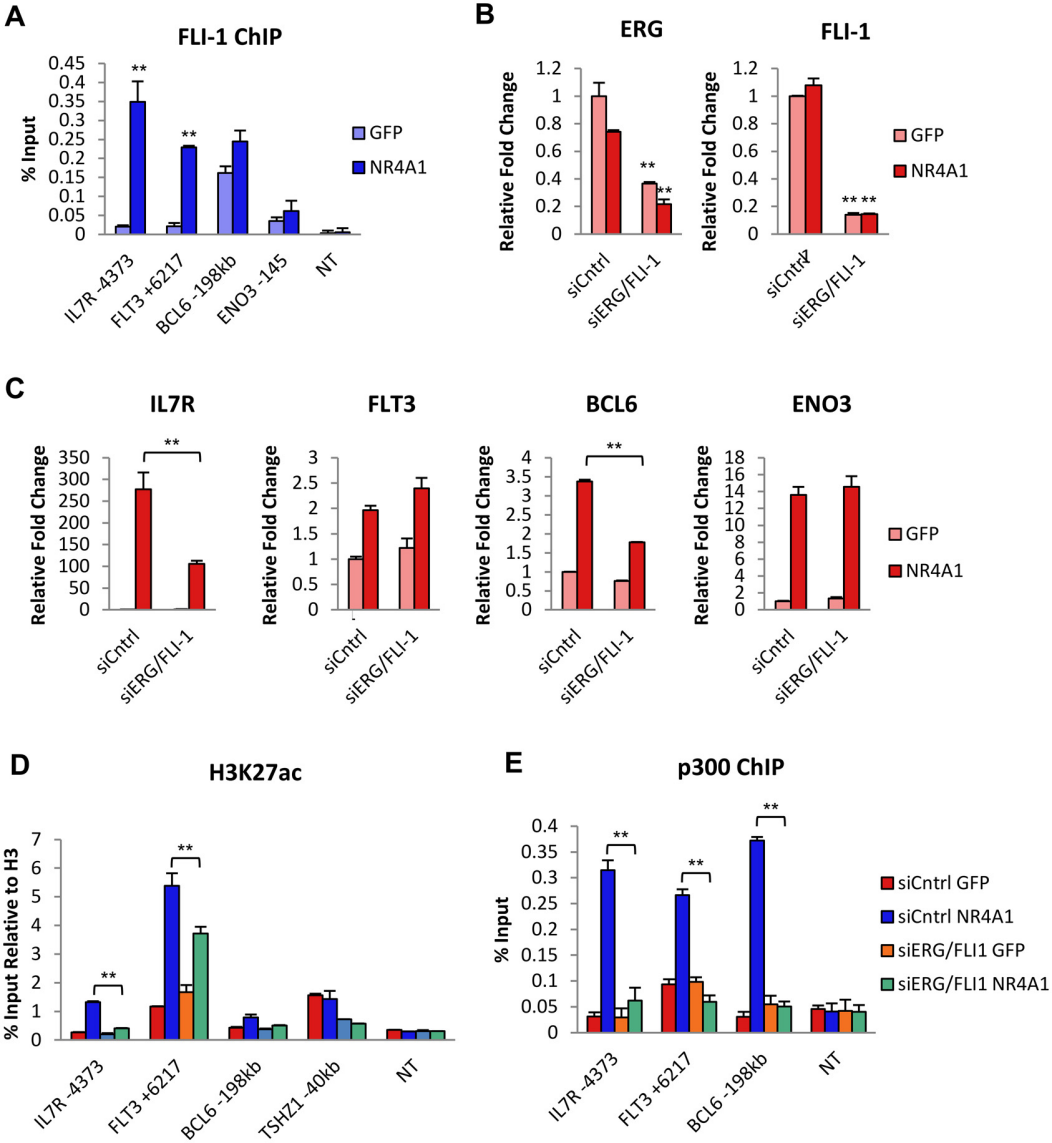
ERG and FLI-1 contribute to NR4A1-mediated enhancer and gene activation via recruitment to NR4A1 binding sites of upregulated genes. (**A**) ChIP-qPCR of FLI-1recruitment to NR4A1 binding sites of upregulated genes 4hr after NR4A1 expression (sample size = 3). (**B**) RT-qPCR analysis of ERG and FLI-1 expression after ERG/FLI-1 siRNA-mediated knockdown at 48hr (sample size = 3). (**C**) RT-qPCR analysis of induced genes at 6hr after 48hr of ERG/FLI-1 knockdown (sample size = 3). (**D**) ChIP-qPCR analysis of H3K27ac at NR4A1 binding sites 48hr after ERG/FLI-1 knockdown (sample size = 3). (**E**) Analysis of p300 recruitment to NR4A1 binding sites 48hr after ERG/FLI-1 knockdown and 4hr of NR4A1 expression using ChIP-qPCR (sample size = 3). NT = non-transcribed region in a gene desert of chr7. Results are means ± SD. **p<0.01, *p<0.05.

Overlap of the NR4A1 targetome with genome wide ERG/FLI-1 occupancy in AML cells revealed that enhancers associated with NR4A1 induced genes were not pre-occupied by ERG/FLI-1 but were nonetheless enriched for the ETS motif. These observations suggested that ETS factors may cooperate with NR4A1 in the activation of NR4A1 bound distal enhancers. AML cells that express the AML-ETO translocation reportedly express high levels of 3 ETS factors: ERG, FLI-1, and TEL [36], which was corroborated by our microarray expression data in Kasumi-1 cells. Since ERG and FLI-1 occupy common genomic sites in AML cells [36], we asked whether NR4A1 can recruit ERG/FLI-1 to distal enhancers of NR4A1-dependent induced genes using ChIP-qPCR to assess FLI-1 occupancy. Consistent with genome wide ERG/FLI-1 genome binding data, this analysis revealed the distal NR4A1 binding regions of *IL7R* and *FLT3* were almost devoid of FLI-1 binding in the absence of NR4A. However, significant recruitment of FLI-1 was observed at both sites in an NR4A1 dependent manner and also enriched at *BCL6* despite a relatively higher apparent basal FLI-1 occupancy at this site (Fig 6A). Notably, NR4A dependent FLI-1 recruitment was not observed at the proximal NR4A1 binding site of the NR4A target gene *ENO3*, indicating that NR4A1-dependent FLI-1 recruitment is also target gene and distal enhancer selective.

**Fig 7:**
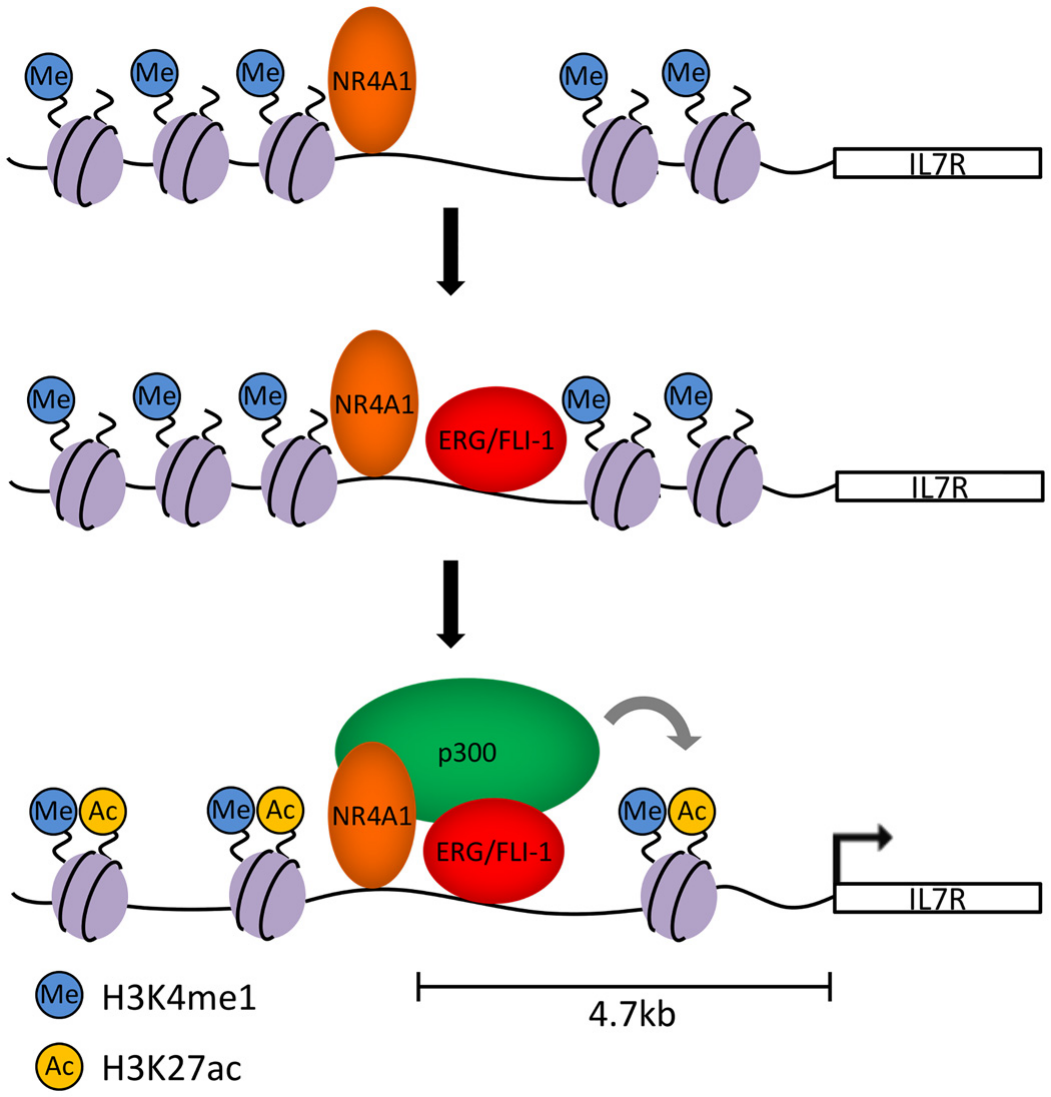
Model of NR4A1-dependent enhancer activation. NR4A1 binds to distal, DNase hypersensitive enhancer elements of upregulated target genes such as IL7R that are marked by H3K4me1. NR4A1 then recruits the ETS transcription factors ERG/FLI-1 which are required for recruitment of the histone acetyltransferase p300 that catalyzes acetylation of H3K27 and results in gene transcription.

To address the functional contribution of ERG/FLI-1 to NR4A1 dependent distal enhancer activation, we then examined the consequences of siRNA mediated codepletion of ERG/FLI-1 on NR4A1-dependent gene induction. Knockdown of ERG and FLI-1 (Fig 6B and 6C) resulted in reduced induction of two of the three genes tested, and consistent with lack of FLI-1 binding to ENO3, ERG/FLI-1 codepletion did not inhibit NR4A1-dependent induction of ENO3 (Fig 6C). Next, we tested ERG/FLI-1 contribution to the acetylation status of H3K27 and also to recruitment of p300. We found that codepletion of ERG/FLI-1 resulted in inhibition of NR4A1 dependent H3K27ac at distal enhancers of all three induced genes (Fig 6D) and also strongly prevented NR4A1 dependent recruitment of p300 (Fig 6E). Together, these data support a model whereby NR4A1 can induce expression of a subset of target genes through reprogramming of ERG/FLI-1 binding to NR4A occupied distal enhancer loci to establish active distal enhancer domains through ERG/FLI-1 dependent recruitment of p300 to enable p300 dependent acetylation and activation of H3K4me1 primed enhancers (Fig 7).

## Discussion

We have previously shown that the AML tumor suppressors, NR4A1 and NR4A3 function redundantly in Kasumi-1 AML-ETO positive human AML cells to inhibit AML cell growth and reprogram a subset of AML gene expression signatures [15]. In the present study, we have used a combination of ChIP-seq analysis of NR4A1 binding to chromatin together with transcription profiling of genes that are acutely regulated by NR4A1 to understand the genome wide transcriptional mechanisms of action of NR4As. Using this approach, we have identified an NR4A1 targetome consisting of 685 genes that are directly regulated by NR4A1. The NR4A1 targetome represents approximately 10% of the 6984 unique genes occupied by NR4A1 in Kasumi-1 cells. The vast majority of genome wide chromatin occupancy by NR4A1 localizes to sites that occur distal to the promoters of NR4A regulated genes in a pattern similar to that previously observed for other nuclear receptors [31,49–51].

Analysis of gene specific mechanisms of transcription by NR4As has revealed that they regulate gene expression via direct binding as monomers to an extended nuclear receptor half-site (NBRE; AAACCGTA) or through homo- or heterodimeric intractions with a Nur response element (NurRE) composed of palindromic repeats of a core AGGTCA motif [52]. Further, in the absence of DNA binding, NR4As can also repress gene transcription by at least two distinct mechanisms. NR4A2 has been shown to repress NFkB activated inflammatory gene programs in macrophages via tethering to NFkB and recruitment of corepressors to NFkB regulated inflammatory genes [2]. NR4A2 can also antagonize ETS mediated gene transactivation in chondrocytes in the absence of DNA binding via unknown mechanisms [53]. Our analysis of transcription factor motifs enriched at NR4A binding sites of both the NR4A cistrome and targetome in AML cells revealed enrichment only for the monomeric NBRE response element, indicating NR4A monomeric interactions with DNA are likely to play a dominant role in this system. We recently demonstrated that both growth inhibition and repression of key NR4A target genes, including *MYC*, require a functional DNA binding domain of NR4A1 and NR4A3 [15]. Consistent with this finding and our observation that NR4A1^CEAA^ does not regulate genes enriched for NFkB target genes, we did not observe enrichment of NFkB motifs within NR4A1-occupied regions of NR4A1 repressed genes despite constitutive NFkB activity in AML [54]. Alternatively, NR4A1 binding sites associated with both induced and repressed genes are highly co-enriched for the NBRE and ETS factor motifs.

Interrogation of the NR4A1 targetome demonstrated that NR4A1 binding nearby upregulated genes is often located in non-DNase hypersensitive regions. We further find that these regions are predefined by H3K4me1 prior to NR4A1 binding. In contrast to pioneering factors such as FOXA1 or PU.1 which facilitate H3K4me1 deposition [55–57], we find no evidence that NR4A1 establishes H3K4me1 at these enhancers. Although numerous studies have found that nuclear receptors often bind to regions of DNaseI hypersensitivity [58,59], the ability of nuclear receptors to bind to non-DHS regions and initiate changes in DHS has recently emerged as an indicator of cell type specific functional enhancers [59–62]. Similarly, the recruitment of p300 to enhancers is also associated with acquisition of DNaseI hypersensitivity [40,41,47,56,63]. Our observation that NR4A1 binds non-DHS sites and increases H3K27 acetylation via recruitment of p300 suggests that NR4A may utilize a similar mechanism of gene activation and warrants further examination of NR4A1 effects on p300 recruitment and differential DHS on a genome wide scale.

The association of nuclear receptors with ETS factor motifs in various hematopoietic cells has previously been reported on a genome wide scale for vitamin D receptor and the orphan nuclear receptor TR4 [64,65], but the importance or functionality of this potential cooperation has not been addressed in detail. However, treatment of t(16;21) AML with all-trans retinoic acid (ATRA) has recently been shown to recruit ERG to new genome-wide binding sites, suggesting that the RARa/RXR heterodimer is capable of reprogramming ERG binding. Conversely, ERG has been reported to indirectly reprogram the androgen receptor cistrome in prostate tumors [66,67]. Our observation that NR4A1-dependent histone acetylation requires expression of the ETS factors ERG/FLI-1 and is associated with NR4A1-dependent recruitment of FLI-1 to the NR4A1 distal primed enhancers at a subset of induced NR4A1 target genes suggests that NR4As may be capable of reprogramming ERG/FLI-1 binding in AML, though this remains to be tested on a global scale.

Recent analysis of genome wide occupancy of ETS factors ERG and FLI-1 in AML-ETO positive SKNO-1 human AML cells has revealed that both factors occupy common genomic regions and that a subset of ERG/FLI-1 occupied enhancers direct the majority of AML-ETO binding for aberrant transcriptional repression of myeloid differentiation genes [36,68–70], Therefore, the enrichment of the ETS and RUNX1 motifs in the NR4A1 targetome could potentially represent antagonism of AML-ETO by NR4A1. However, we find that NR4A1 binding sites in Kasumi-1 cells minimally overlap with AML-ETO binding sites identified in SKNO-1 cells [35] (data not shown). Furthermore, these results are consistent with our observation that NR4A1 does not induce myeloid differentiation gene programs and, therefore, does not likely play an opposing role to the anti-differentiation transcriptional profile observed for AML-ETO [68–70]. In contrast, we observe that NR4A1 regulates a transcriptional profile dominated by proliferative genes, specifically a core MYC oncogenic signature, which is likely the primary mechanism of tumor suppression by NR4As.

Interestingly, analysis of the ERG cistrome in human CD34+ hematopoietic progenitors previously identified the nuclear receptor half site as a coenriched motif with ERG binding sites [36], suggesting that nuclear receptors may cooperate with ERG in non-AML hematopoietic progenitor populations, and we and others have previously shown that NR4As are highly expressed within this population [10,11]. Together, these data have implications during early hematopoiesis where NR4As may also cooperate with ETS factors to maintain appropriate cell fate decisions by responding to a primed enhancer landscape.

## Supporting Information

S1 Fig NR4A1 ChIP-seq binding peaks are reproducible. NR4A1 binding profiles illustrating reproducibility of NR4A1 ChIP-seq peaks at *ENO3*. The y axis represents cumulative tag counts across the region.
(PDF)

S2 Fig NR4A1 tumor suppressive transcriptional profiles require DNA binding ability. (**A**) Heatmap of differentially expressed genes 6hr after GFP, NR4A1^WT^ or NR4A1^CEAA^ IVT-mRNA transfection. (**B**) Gene set enrichment analysis (GSEA) of differentially expressed genes. (**C**) Gene ontology annotations generated by DAVID functional annotation of genes induced or repressed by NR4A1^WT^. (**D**) Gene ontology annotations of genes induced or repressed by NR4A1^CEAA^.
(PDF)

S3 Fig NR4A1 directly induces genes involved in cell differentiation. (**A**) Genes included in lymphocyte differentiation annotation derived from NR4A1-induced genes. (**B**) NR4A1 ChIP-seq binding profiles at validated induced genes in Kasumi-1 cells. Arrows denote binding regions validated by ChIP-qPCR. The Refseq transcript for each gene is shown below each locus. The y axis represents cumulative tag counts at each region.
(PDF)

S4 Fig NR4A1 directly represses cell proliferation genes. (**A**) Genes included in the cell proliferation annotation derived from NR4A1-repressed genes. (**B**) NR4A1 ChIP-seq binding profiles in Kausmi-1 cells at validated repressed genes. Arrows denote binding regions validated by ChIP-qPCR. The Refseq transcript for each gene is shown below each locus. The y axis represents cumulative tag counts at each region.
(PDF)

S1 Table Primer sequences used for ChIP-qPCR analysis. (PDF)
