# Supplementary figures and images for: Genome Wide Mapping of NR4A Binding Reveals Cooperativity with ETS Factors to Promote Epigenetic Activation of Distal Enhancers in Acute Myeloid Leukemia Cells
Source: PLoS One. 2016 Mar 3;11(3):e0150450. doi: 10.1371/journal.pone.0150450 (PMC4777543; doi:10.1371/journal.pone.0150450)

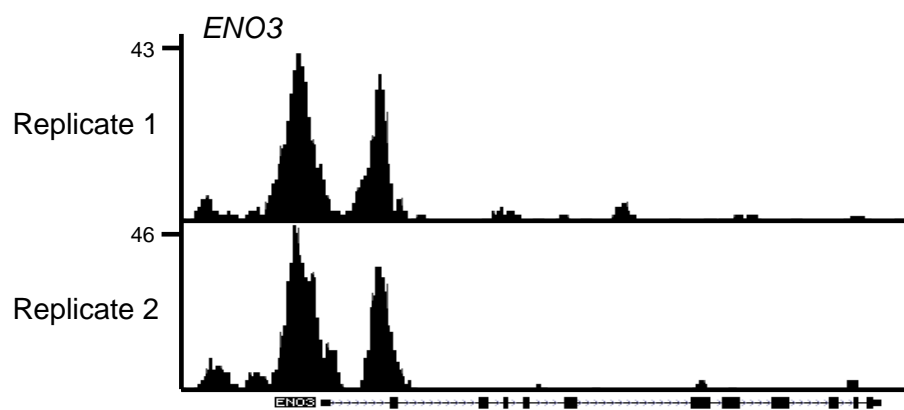

Supplement: S1 Fig — NR4A1 binding profiles illustrating reproducibility of NR4A1 ChIP-seq peaks at ENO3. The y axis represents cumulative tag counts across the region. (PDF) [file pone.0150450.s001.pdf]
