# Supplementary material for: Genome Wide Mapping of NR4A Binding Reveals Cooperativity with ETS Factors to Promote Epigenetic Activation of Distal Enhancers in Acute Myeloid Leukemia Cells
Source: PLoS One. 2016 Mar 3;11(3):e0150450. doi: 10.1371/journal.pone.0150450 (PMC4777543; doi:10.1371/journal.pone.0150450)
Supplement: S2 Fig — (A) Heatmap of differentially expressed genes 6hr after GFP, NR4A1WT or NR4A1CEAA IVT-mRNA transfection. (B) Gene set enrichment analysis (GSEA) of differentially expressed genes. (C) Gene ontology annotations generated by DAVID functional annotation of genes induced or repressed by NR4A1WT. (D) Gene ontology annotations of genes induced or repressed by NR4A1CEAA. (PDF) [file pone.0150450.s002.pdf]

A

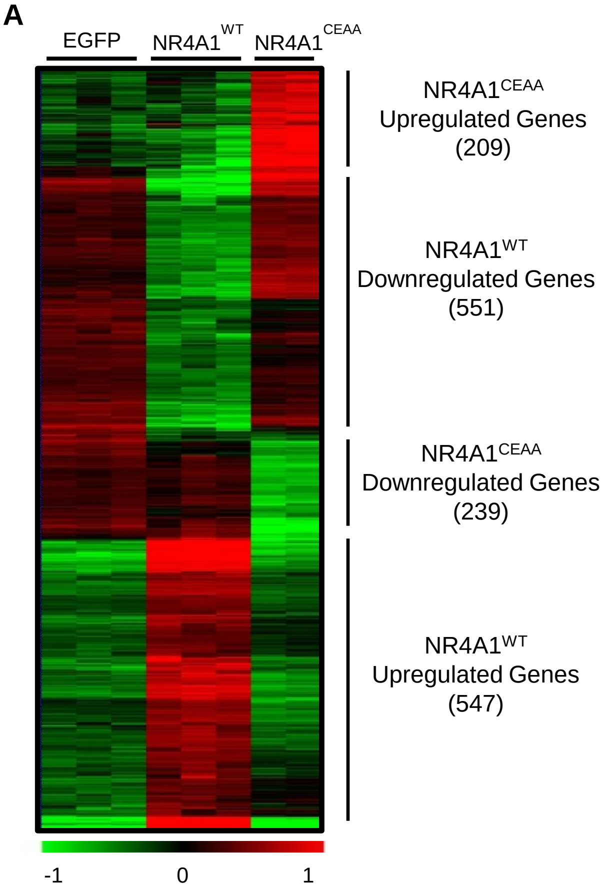

B

NES = -2.37
NOM p-value < 0.000

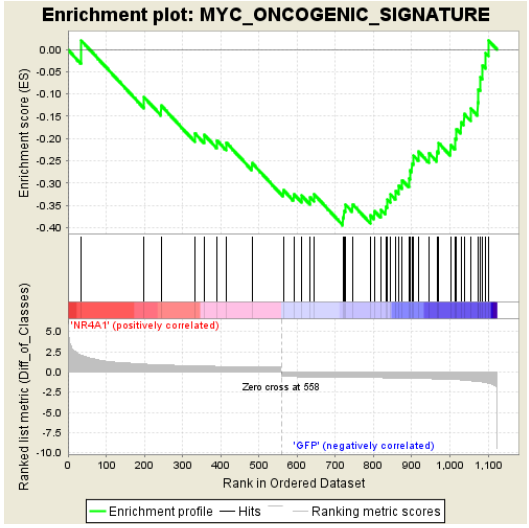

C

Genes induced by NR4A1^{WT}

Biological Process	P value
Lymphocyte Differentiation	1.10E-04
Negative Regulation of Transcription	3.60E-04
Positive regulation of apoptosis	7.20E-04
Dephosphorylation	1.00E-03

Genes repressed by NR4A1^{WT}

Biological Process	P value
Ribosome biogenesis	1.50E-07
ncRNA processing	4.00E-04
Carboxylic Acid Transport	2.00E-03
Cell proliferation	3.30E-03

D

Genes induced by NR4A1^{CEAA}

Biological Process	P value
Ribonucleotide binding	3.60E-08
Cell cycle	2.20E-07
Proto-oncogene	8.20E-06

Genes repressed by NR4A1^{CEAA}

Biological Process	P value
Small GTPase signal transduction	1.70E-04
Actin cytoskeleton organization	7.20E-04
Endomembrane system	1.20E-03
