# Supplementary material for: Genome Wide Mapping of NR4A Binding Reveals Cooperativity with ETS Factors to Promote Epigenetic Activation of Distal Enhancers in Acute Myeloid Leukemia Cells
Source: PLoS One. 2016 Mar 3;11(3):e0150450. doi: 10.1371/journal.pone.0150450 (PMC4777543; doi:10.1371/journal.pone.0150450)

A

Gene Name	Gene Symbol
Interleukin 7 receptor	IL7R
B-cell CLL/lymphoma 6	BCL6
fms-related tyrosine kinase 3	FLT3
IKAROS family zinc finger 1	IKZF1
Histone deacetylase 9	HDAC9
ATPase, Cu++ transporting, alpha peptide	ATP7A
Integrin, beta 1	ITGB1
Interleukin 15	IL15
Protein tyrosine phosphatase, non-receptor 22	PTPN22
Protein tyrosine phosphatase, receptor type, C	PTPRC
Signal transducer and activator of transcription 5A	STAT5A

B

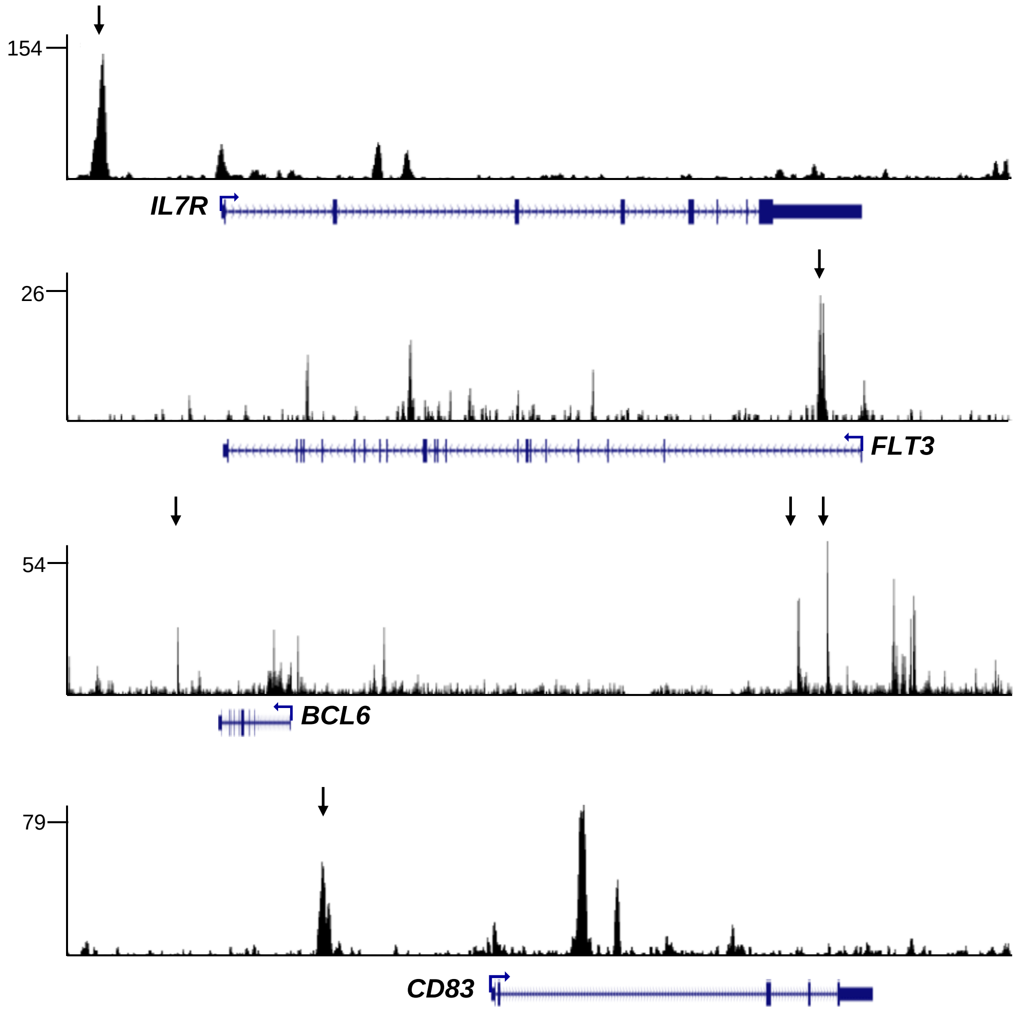

Supplement: S3 Fig — (A) Genes included in lymphocyte differentiation annotation derived from NR4A1-induced genes. (B) NR4A1 ChIP-seq binding profiles at validated induced genes in Kasumi-1 cells. Arrows denote binding regions validated by ChIP-qPCR. The Refseq transcript for each gene is shown below each locus. The y axis represents cumulative tag counts at each region. (PDF) [file pone.0150450.s003.pdf]
