# Supplementary material for: Genome Wide Mapping of NR4A Binding Reveals Cooperativity with ETS Factors to Promote Epigenetic Activation of Distal Enhancers in Acute Myeloid Leukemia Cells
Source: PLoS One. 2016 Mar 3;11(3):e0150450. doi: 10.1371/journal.pone.0150450 (PMC4777543; doi:10.1371/journal.pone.0150450)

A

Gene Name	Gene Symbol
v-myc myelocytomatosis viral oncogene homolog	MYC
B-cell CLL/lymphoma 2	BCL2
Colony stimulating factor 1 receptor	CSF1R
Core binding factor, runt domain, alpha subunit 2	CBFA2T3
v-ets erythroblastosis virus E26 oncogene homolog	ERG
T box 1	TBX1
Bridging integrator 1	BIN1
Dyskeratosis congenita 1	DKC1
Guanine nucleotide binding protein, gamma 2	GNG2
Heme oxygenase 1	HMOX1
Insulin-like growth factor binding protein 4	IGBP4
Megakaryocyte-associated tyrosine kinase	MATK
Patched homolog 1	PTCH1
Pyridoxal (pyridoxine, vitamin B6) kinase	PDXK
Retinoid X receptor alpha	RXRA
Solute carrier family 29, member 2	SLC29A2
Zinc finger protein 36, C3H type-like 2	ZFP36L2

B

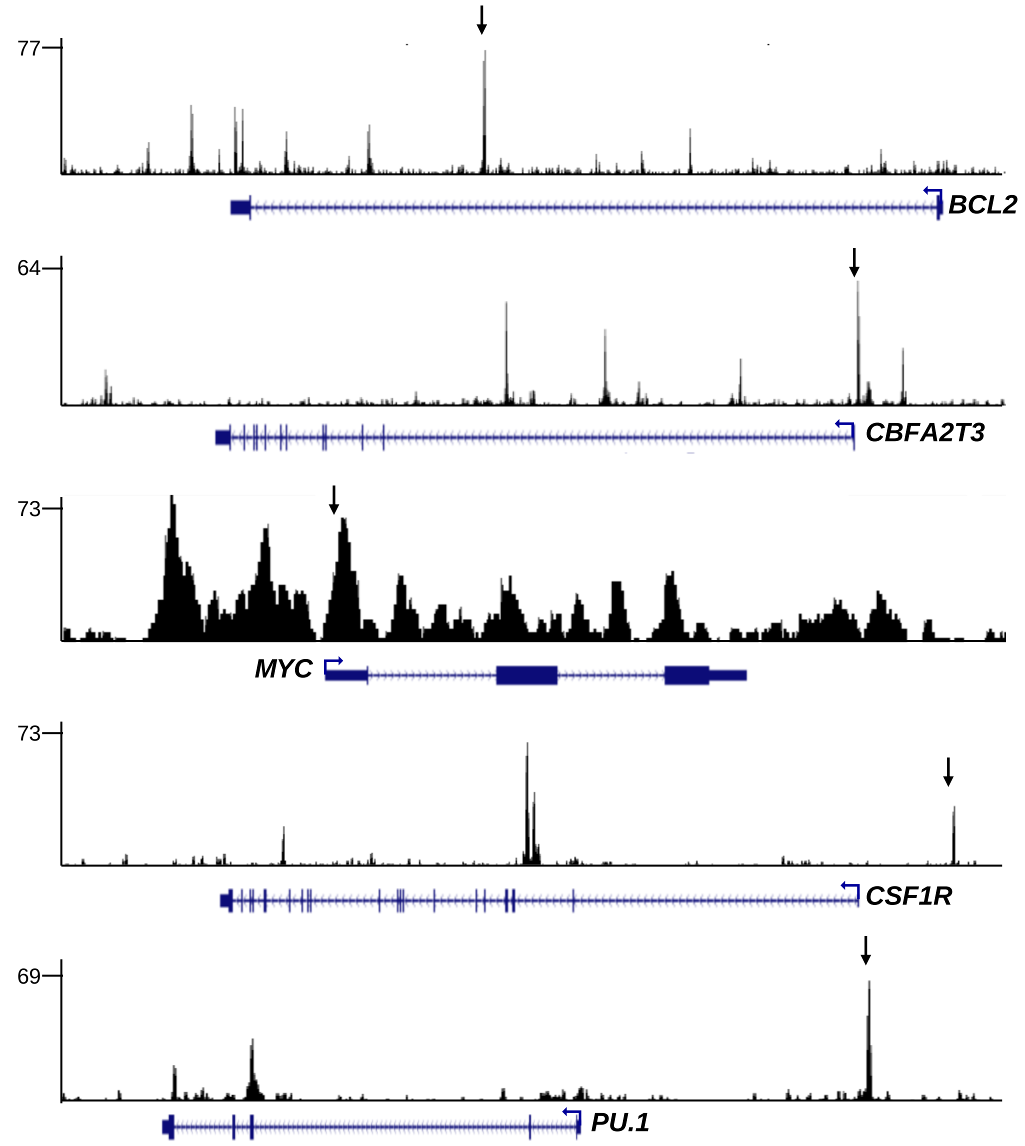

Supplement: S4 Fig — (A) Genes included in the cell proliferation annotation derived from NR4A1-repressed genes. (B) NR4A1 ChIP-seq binding profiles in Kausmi-1 cells at validated repressed genes. Arrows denote binding regions validated by ChIP-qPCR. The Refseq transcript for each gene is shown below each locus. The y axis represents cumulative tag counts at each region. (PDF) [file pone.0150450.s004.pdf]
