# Supplementary material for: Genome Wide Mapping of NR4A Binding Reveals Cooperativity with ETS Factors to Promote Epigenetic Activation of Distal Enhancers in Acute Myeloid Leukemia Cells
Source: PLoS One. 2016 Mar 3;11(3):e0150450. doi: 10.1371/journal.pone.0150450 (PMC4777543; doi:10.1371/journal.pone.0150450)
Supplement: S1 Table — (PDF) [file pone.0150450.s005.pdf]

Name	Sequence
IL7R FWD	5' TGCATCCAGAGACAGAGGCTAAA
IL7R REV	5' TGGGCAGAGTTTGAAATGTCAT
FLT3 FWD	5' CATTATAAGGTCAATTGGTAGCCAAGA
FLT3 REV	5' CCAAGACCGAATTCAGTGACTATCT
BCL6 -207kb FWD	5' CACGTCTCTCGTGCATTATGC
BCL6 -207kb REV	5' GGAGCAGAGGAAGATGTAGGTCAT
BCL6 -198kb FWD	5' TCCCATTAGGGCCCAGATC
BCL6 -198kb REV	5' AGCCCTTTGAGATTAGCCTCTCTAG
BCL6 +38kb FWD	5' TTGTGACTTTTGAGGGTAGCTCAT
BCL6 +38kb REV	5' GGCTGGTGTCACAAGAGAATCA
CD83 FWD	5' CCCCGCCTTCCCCTTT
CD83 REV	5' GACTTCCTGGCTGGACTGATCT
BCL2 FWD	5' TGGAATACCTTGCCCCAGATT
BCL2 REV	5' AAGGTCATGTGTTATCTGAGAAGCA
CBFA2T3 FWD	5' GGGCCGAGGAAACACATG
CBFA2T3 REV	5' CATTCCGGTTTGACCTTCCA
MYC FWD	5' GCTTGGCGGGAAAAAGAAC
MYC REV	5' CCCGAAAACCGGCTTTTATA
CSF1R FWD	5' ACCCGTCTTGTGACCTTTGC
CSF1R REV	5' TGAGGCTACCAAAATGACAGTTAGA
PU.1 FWD	5' AGTGGTGGCCCGAGTTTTC
PU.1 REV	5' TCTTGGCGGAAGCTGTTAGG
NT FWD	5' AACCTCACTTTCATTGTTACTAGCCATA
NT REV	5' CGCTCAAGGATGTCAGTAGCAT
p15INK4b FWD	5' TCGTCGCTTGCACATCCTCTCTTA
p15INK4b REV	5' CGCATGGCAAGTCACATGCGTAAA
TSHZ1 FWD	5' ACATCTCCCTGGCCAAAGC
TSHZ1 REV	5' CCTGCGCCCTCTGAAGGTA
